# A ribosome-related signature in peripheral blood CLL B cells is linked to reduced survival following treatment

**DOI:** 10.1038/cddis.2016.148

**Published:** 2016-06-02

**Authors:** T Sbarrato, E Horvilleur, T Pöyry, K Hill, L C Chaplin, R V Spriggs, M Stoneley, L Wilson, S Jayne, T Vulliamy, D Beck, I Dokal, M J S Dyer, A M Yeomans, G Packham, M Bushell, S D Wagner, A E Willis

**Affiliations:** 1 Medical Research Council Toxicology Unit, Hodgkin Building, PO Box 138, Lancaster Rd, Leicester LE19HN, UK; 2 The Babraham Institute, Babraham, Cambridge, UK; 3 Department of Cancer Studies, Ernest and Helen Scott Haematology Research Institute, University of Leicester, Lancaster Road, Leicester LE1 7H, UK; 4 Centre for Genomics and Child Health, Blizard Institute, Barts and The London School of Medicine and Dentistry, 4 Newark Street, Whitechapel, London E1 2AT, UK; 5 Cancer Research UK Centre, Faculty of Medicine, University of Southampton, Southampton, UK

## Abstract

We have used polysome profiling coupled to microarray analysis to examine the translatome of a panel of peripheral blood (PB) B cells isolated from 34 chronic lymphocytic leukaemia (CLL) patients. We have identified a ‘ribosome-related' signature in CLL patients with mRNAs encoding for ribosomal proteins and factors that modify ribosomal RNA, e.g. *DKC1* (which encodes dyskerin, a pseudouridine synthase), showing reduced polysomal association and decreased expression of the corresponding proteins. Our data suggest a general impact of dyskerin dysregulation on the translational apparatus in CLL and importantly patients with low dyskerin levels have a significantly shorter period of overall survival following treatment. Thus, translational dysregulation of dyskerin could constitute a mechanism by which the CLL PB B cells acquire an aggressive phenotype and thus have a major role in oncogenesis.

Chronic lymphocytic leukaemia (CLL) is characterised by the accumulation of small monoclonal B cells in the peripheral blood (PB), lymph nodes (LN) and bone marrow (BM). The circulating CLL cells in PB are largely arrested in the G0/G1 phase of the cell cycle; however, they undergo spontaneous apoptosis *in vitro*.^1^ Several studies, particularly those using heavy water, have also reported that a significant amount of CLL B-cell proliferation occurs in the pseudofollicles. Both proliferation and resistance to apoptosis are believed to be governed by supporting stromal cells in the tissue microenvironment, and the disease is characterised by a dynamic imbalance between proliferation and apoptosis of neoplastic B-lymphocytes-coexpressing CD5 and CD19 antigens.

CLL has an extremely variable outcome with overall survival (OS) ranging from months to decades; thus, some patients require immediate treatment upon diagnosis, whereas others remain asymptomatic for the whole course of the disease. The sub-classification of CLL patients into groups based on a number of criteria has aided the prediction of disease outcome. For example, two early pivotal studies demonstrated that cases where the malignant B cells expressed somatically hypermutated immunoglobulins had a good prognosis, whereas expression of unmutated immunoglobulins was associated with relatively aggressive disease.^2, 3^ In later studies, cDNA microarray analysis was used to identify gene signatures related to a number of molecular subtypes of the disease^4, 5^ and pathways associated with disease evolution.^6^ Finally, whole-exome sequencing,^7^ high-density methylation microarrays^8^ and RNA sequencing^9^ have allowed clustering of patients into additional molecular subgroups, independent of immunoglobulin heavy-chain variable region (IGVH) status, which, importantly, display different outcomes in terms of long-term survival.^9^

However, changes at the level of transcription are not necessarily linked to protein expression, which can also be controlled by regulating translation; a three-stage process comprised of initiation, elongation and termination, where initiation is considered to be the rate-limiting step.^10^ Initiation can be controlled through changes in the expression or activity of components of eukaryotic initiation factor (eIF) 4F complex (comprised of eIF4E, the cap-binding protein; eIF4A1, a DEAD box helicase and eIF4G, a scaffold protein). There are many examples to show that aberrant translational control can contribute to the aetiology and progression of cancer.^11^ For example, altered expression/phosphorylation of eIF4E and eIF4B (which stimulates the helicase activity of eIF4A) are associated with poor prognosis in diffuse large B-cell lymphoma.^12, 13^ In CLL signalling in the LN microenvironment promotes tumour cell proliferation^14^ and two recent studies have shown the translational machinery is important in this context.^15, 16^ Thus stimulation of PB CLL B-cells *in vitro* by either CD40L-expressing stromal cells or the B-cell receptor (BCR) promotes translation by stimulating eIF4F complex assembly or expression of eIF4G and eIF4A1.^15, 16^ Following stimulation of the BCR, it has been shown that c-Myc protein levels are increased as a consequence of translation stimulation in CLL;^15^ however, the full repertoire of the mRNAs (the translatome) that are controlled at this level has yet to be defined.

The ribosome is also important in disease progression and defects in the ribosome biogenesis pathway are also associated with an increased cancer risk. For example, a group of rare disorders termed ‘ribosomopathies', which have mutations in genes encoding for ribosomal proteins or ribosome maturation factors, have an increased risk of developing leukaemias and solid tumours.^17^ Thus, individuals with Diamond–Blackfan anaemia with mutations in ribosomal proteins, for example, ribosomal protein small (RPS)-19, have a 28-fold higher incidence of acute myeloid leukaemia than the general population.^18^ Somatic mutations have also been identified in ribosomal proteins in cancers, and mutations in ribosomal protein large (RPL)-5 and RPL11 have been found in patients with T-cell acute lymphoblastic leukaemia (T-ALL),^19^ and in RPL10 and RPL22 in gastric and ovarian cancers,^20, 21^ and RPL15 and RPS15 have been identified recently as mutated in a subset of CLL patients.^22, 23^

Despite previous studies on translation status in CLL following *in vitro* stimulation, neither the translatome nor the role of the ribosome has been examined in circulating CLL B-cells. Therefore, in this study, the translatome of PB CLL B cells was identified in B cells isolated directly from 34 patients and three normal donors by carrying out polysome profiling coupled to cDNA microarray. Our data show that there is a ribosome-related signature in a PB CLL B-cells with reduced polysomal association and expression of ribosomal proteins, and factors that modify ribosomal rRNA, including *DKC1* that encodes for the highly conserved nucleolar protein dyskerin. The latter protein associates with the H/ACA class of small nucleolar RNAs and functions as a pseudouridine synthase, converting uridine to pseudouridine residues in ribosomal RNA (rRNA) during ribosomal maturation in the nucleolus. Importantly, we show that *DKC1* protein expression is a prognostic factor correlating with poor OS following treatment.

## Results

### Translational profiling of CLL patient samples

**Figure 1:**
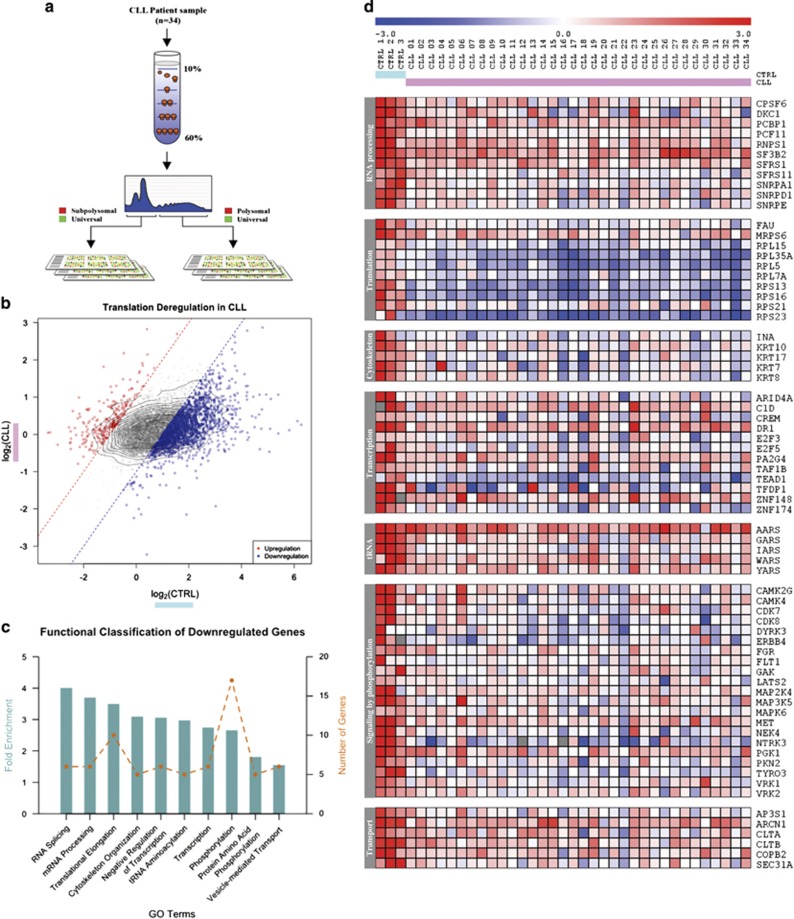
Polysome profiling of CLL patients identified a dysregulation of translation, especially within a group of ribosome-related genes. (**a**) General workflow of polysome profiling. mRNAs from CLL patients or healthy controls were separated in a sucrose density gradient and analysed by spectrophotometry. Pooled fractions of subpolysomal and polysomal regions were hybridised to cDNA microarrays along with an universal RNA control. (**b**) Represents the distribution of the translational ratios for all genes between CTRL (*x* axis) and CLL (*y* axis; expressed as log2(polysomal/subpolysomal)). Dotted lines represent the fictive border for fold change of 2 and 0.5 (red and blue, respectively). (**c**) Gene functional classification plot showing top-enriched clusters using gene ontology terms (biological processes). Under a high stringency setting, only the downregulation list yielded significant enrichment. Dotted line represents the number of genes in each cluster. (**d**) Heatmap of genes in the enriched functional groups for the B-cell controls (cyan) and CLL patients (pink)

To study the translational status of PB CLL purified B cells isolated from patient samples, polysome profiling on cDNA microarrays was performed and the data compared with control B cells (CD45+, CD19+ and CD3−) obtained using CD20+ selection. This subpopulation of B cells was chosen, as large numbers of cells were required, and moreover it allowed the comparison of our data sets with previous studies.^39^ Cytoplasmic lysates prepared from freshly isolated PB CLL B cells from 34 patients or three controls were separated on a 10–60% sucrose gradient. RNA derived from fractions 1–5 (subpolysomal region) and fractions 6–10 (polysomal region) were compared on cDNA microarrays against a commercial universal RNA as internal reference for normalisation (Figure 1a). Intensity signals for the subpolysomal and polysomal composition were then used to identify mRNAs, preferentially associated with actively translating ribosomes in CLL patients. In brief, the data was background corrected and normalised to a universal RNA control to extract the logged ratio of polysomal over subpolysomal signals (Figure 1a). The identification of significantly dysregulated genes was performed using four different statistical tests (Limma, Rankprod, SAM and *t*-test). To limit the number of false positives identified in each statistical test, a gene was retained as candidate for deregulation if it was significantly identified in three out of four tests.

On average 36% fewer mRNAs were polysomally associated in CLL when compared with the controls; 1746 mRNAs showed a decrease in polysomal association relative to the controls, compared with only 227 mRNAs that showed a significant increase (Figure 1b). Interestingly, changes in translatome did not correlate with IGVH mutational status (Supplementary Figure 1).

Gene functional classification algorithm was applied to the list of identified candidates to classify genes into functional groups for the translational signature in PB CLL B cells (Supplementary Figure 2; Figure 1c and d). The groups of mRNAs that show reduced polysomal association included those that encode proteins that function in RNA binding, a large subset of ribosomal proteins and dyskerin that modifies rRNA (Figure 1c and d; Supplementary Figure 2b and c). This functional cluster of genes (enrichment score of 3.5) inferred that a common pattern of translational regulation existed across the PB CLL patients studied. Importantly, analysis of two transcriptional data sets did not identify any enrichment in GO terms for ribosome or protein synthesis (Supplementary Figure 3).

### There is a decrease in expression of eukaryotic initiation factors and ribosomal proteins in CLL

**Figure 2:**
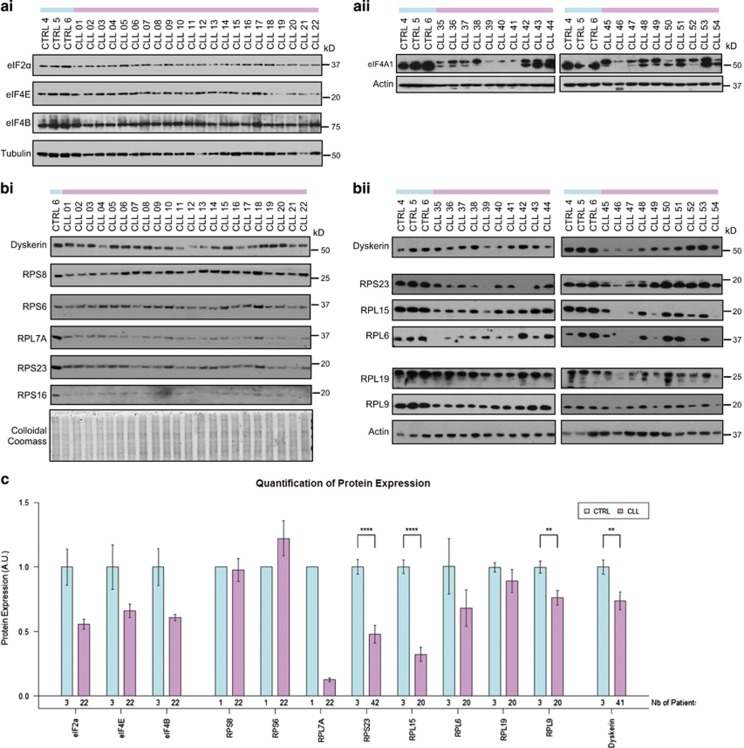
Immunoblot analysis to confirm downregulation mRNA expression by microarray results in reduced protein expression. B-cell lysates from CLL patients or healthy controls were subjected to SDS-PAGE and western blot analysis. (**ai**) and (**aii**) show expression of proteins involved in translation initiation. (**ai**) and (**bii**) show expression of ribosomal proteins/proteins involved in ribosome maturation. Patients samples used for (**ai**) and (**bi**) were from the same cohort as the samples used in the microarray in Figure 1, whereas another cohort was used to produce (**aii**) and (**bii**). Tubulin and colloidal coomassie staining were used as loading controls for western samples for (**ai**) and (**bi**), where actin was used for (**aii**) and (**bii**). (**c**) Western blots from **a** and **b** were quantified and the mean expression levels were plotted for controls and CLL patients. Error bars show the S.E.M. Significance was assessed by Student's *t*-test (**P<0.01, ****P<0.0001)

Western blot analysis was carried out on the proteins that corresponded to a subset of mRNAs that were identified as less polysomally associated to validate the microarray data, using the CLL patient samples used for microarray analysis, in addition to 40 further patient samples. Three B-cell samples derived from healthy individuals were used as controls. The data confirmed that components of the translational machinery identified in the translational profile as less polysomally associated, namely, eIF4B and eIF2 alpha, showed decreased expression relative to the controls, in addition to eIF4E (Figure 2ai). Although the overall levels of eIF4A appeared similar in all the CLL patients, a significant proportion of this protein migrated at a higher molecular weight. There is no evidence in data bases to support an alternatively translated form of eIF4A1 and therefore the most likely explanation is that in these cells eIF4A1 contains a protein modification (Figure 2aii), which could be sumolyation, as described.^40^ However, the precise type of modification has yet to be identified. Ribosomal protein expression was also examined and the data again confirmed the array data, with significant decreases in expression of ribosomal proteins, including RPS23, RPL7A, RPL9 and RPL15 (Figure 2bi, bii and c). Interestingly, dyskerin a nucleolar protein that is involved in post-transcriptional modifications of rRNA also exhibited decreased expression in PB CLL B cells compared with normal B cells (Figure 2bi, bii and c). Although the overall levels are ribosomal proteins and dyskerin expression are significantly decreased in CLL there is variation between patients. For example, CLL18 has expression of dyskerin and ribosomal proteins that is comparable to the control samples (Figure 2bi).

### Protein analysis confirms a decrease in dyskerin levels

**Figure 3:**
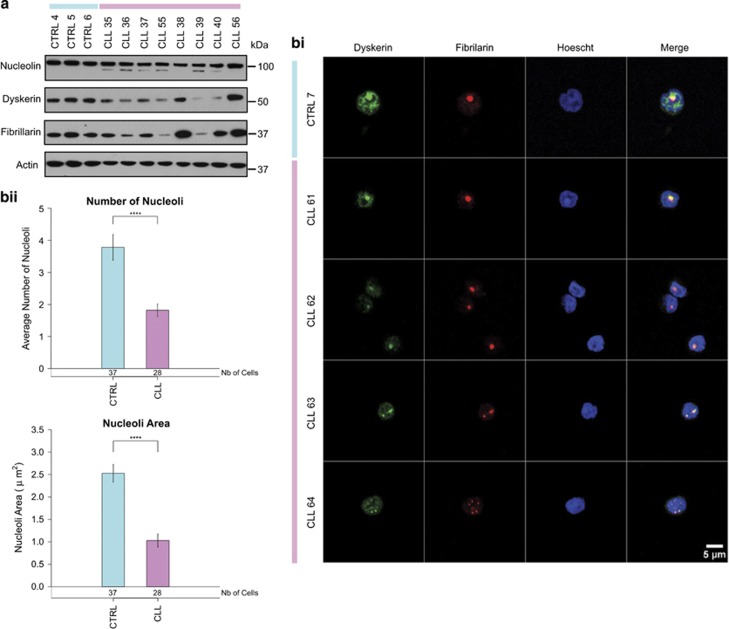
Nucleoli are smaller in CLL cells. (**a**) Western blot showing expression of nucleolin and fibrillarin in CLL samples and control B cells. (**bi**) Immunofluorescence using fibrillarin and dyskerin antibodies. Cells representative of one control and three CLL samples are shown. (**bii**) Fibrillarin immunofluorescence was quantified to estimate the average size and number of nucleoli in CLL and control cells. The plot represents average ± S.E.M., the number of cells studied is indicated at the bottom of the bar. Significance was assessed by Student's *t*-tests (****P<0.0001)

Decreased levels of dyskerin and ribosomal proteins prompted us to examine other nucleolar proteins. Fibrillarin exhibited the same decreased expression, and more importantly its expression showed similar variation to dyskerin protein levels between patients (Figure 3a). It has been shown previously that in CLL there is aberrant localisation into the cytoplasm of proteins that have a role in ribosome biogenesis, for example, nucleolin and nucleophosmin-1.^41, 42^ Therefore, immunofluorescence studies were performed to determine whether dyskerin was similarly affected. B cells derived from a panel of four CLL patients were used in conjunction with a B-cell control, purified from a healthy donor (Figure 3bi). In CLL B-cells dyskerin co-localises with fibrillarin in the nucleoli, with no detectable cytoplasmic expression. Moreover, the immunofluorescence data confirmed the reduced expression of this protein in CLL (Figure 3a). Importantly, there was a significant reduction in size and number of nucleoli overall (Figure 3bii), consistent with the decreased expression of ribosomal proteins (Figure 2) and a reduced number of ribosomes.

It has been shown previously that reduced levels of dyskerin and, as a consequence, decreased rRNA pseudouridylation, affects ribosome–ligand interaction and translational fidelity in both yeast and human systems.^43^ Moreover, in addition to modification of rRNA and TERC by dyskerin,^44^ recent data also suggest that specific subsets of mRNAs may be pseudouridylated, including those encoding ribosomal proteins.^45^

### Aberrant ribosome biogenesis in CLL

**Figure 4:**
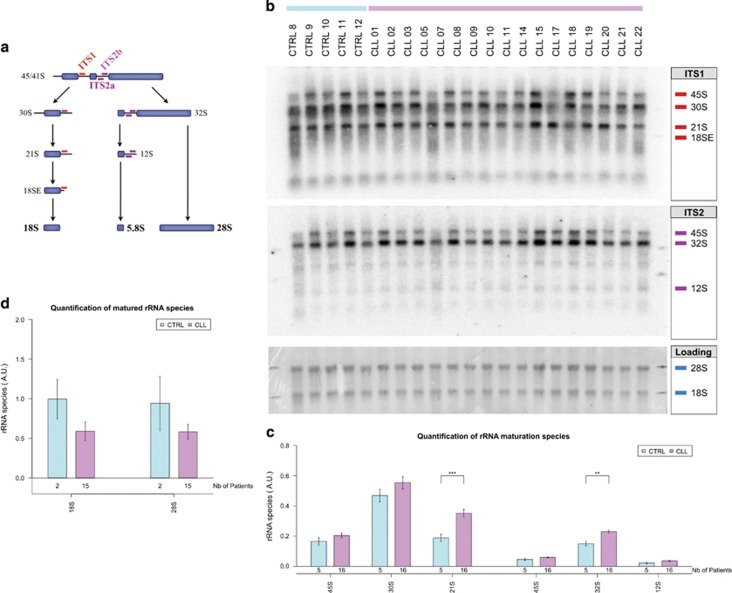
Maturation of ribosomal RNA is altered in CLL cells. (**a**) Simplified schematic overview of the pre-rRNA processing pathways. Internal transcribed spacer (ITS) regions are shown. (**b**) Northern blot analysis with ITS1 and ITS2 probes (18S and 28S, respectively) were used to detect different pre-rRNA species. Methylene blue staining of the total rRNA was used as a loading control (bottom panel). (**c**) Quantification of the pre-rRNA species from (**b**). (**d**) Total RNA signal from quantitative PCR for matured rRNA 18S and 28S for two B-cell controls and 15 CLL patients. S.E.M. are represented and significance was assessed by Student's *t*-tests (**P<0.01 and ***P<0.001)

Impaired pseudouridylation conversion and a decreased synthesis of ribosomal proteins, may have a direct impact on rRNA maturation and processing in CLL cells. Thus, the different forms of rRNA species produced during the process of maturation were studied by northern blotting in a panel of 16 CLL patients complemented with five normal counterpart B-cell controls. Two small oligonucleotide probes complementary to the ITS1 and ITS2 allow the detection of the 45S/41S, 30S, 21S and 18S-E forms with the ITS1 probe and the 45S/41S, 32S and 12S forms with the ITS2 probe (Figure 4a and b). The data showed no specific blockage site in the rRNA maturation pattern; however, the quantification showed a slight increase in all pre-rRNA species in the CLL samples compared with the control B-cell samples (Figure 4c). This could imply that the rRNA maturation is slowed down in the CLL patients presumably because of decreased pseudouridylation in the rRNA. It was shown using *DKC1*-mutant cell line that non-pseudouridylated rRNA processing was delayed and resulted in reduced amounts of matured 18S and 28S rRNAs compared with the wild-type cell.^46^ As such, total levels of matured 18S and 28S rRNAs were markedly reduced in CLL patients compared with control B cells in our study (Figure 4d), thus confirming an aberrant ribosome biogenesis in CLL.

Circulating CLL cells are known to be essentially quiescent, so it was possible that reduced amounts of rRNA and proteins, as well as low dyskerin levels could simply reflect the fact that quiescent cells have lower translation rates. To address this point, we purified B cells from five different CLL patients and cultivated them in presence of anti-IgM antibody to mimic activating conditions in the lymph nodes. Interestingly, dyskerin levels did not change in activated cells, suggesting that low expression of dyskerin is not related to quiescence and could be a specific feature of CLL (Supplementary Figure 4).

### Patients with reduced expression of DKC1 display defects in ribosomal protein expression

**Figure 5:**
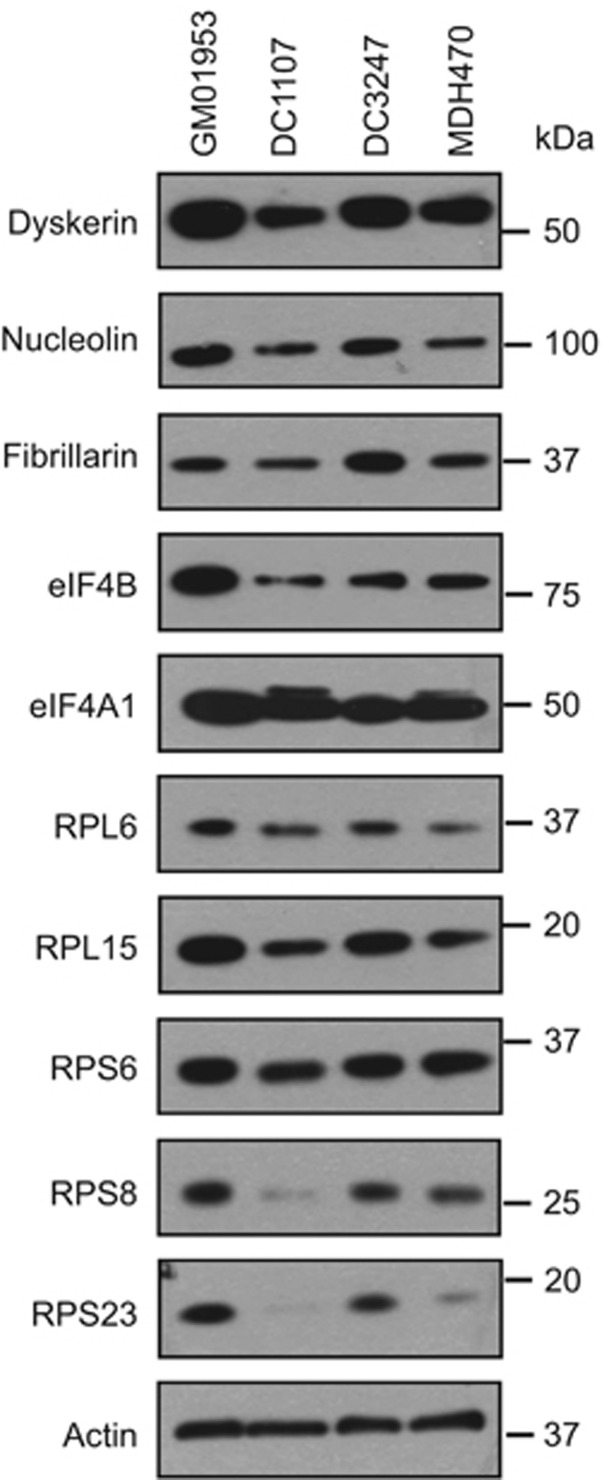
Ribosomal proteins are downregulated in dyskeratosis congenita patient cell lines. Western blot analysis showing proteins expression in lymphoblastoid cell lines derived from three dyskeratosis congenita patients (DC1107, DC3247 and MDH470) and one healthy control (GM01953)

To investigate whether we could recapitulate our data by reducing dyskerin expression, a siRNA-based approach was employed in GM01953, a control B-cell line. However, in this system the reduced dyskerin levels were only maintained for 72 h, which was not long enough to observe any effect on ribosomal proteins, most probably because of their long half-life (data not shown). In support of these data, it has been shown recently that transient depletion of *DKC1* does not affect ribosome composition.^47^ Therefore, to investigate whether there was a correlation between reduced *DKC1* expression and synthesis of translation machinery, three cell lines derived from patients with dyskeratosis congenita that had a mutation in *DKC1* gene were used as an alternative. Western blot analysis was performed and data show that there was a significant decrease in the expression of RPS8, RPS23, RPL6, RPL15 and RPL19. In addition, there was a decrease in expression of eIF4B and interestingly the high migrating form of eIF4A was also observed (Figure 5 and 2aii). Taken together these data suggest a link between aberrant *DKC1* function and reduced expression of some ribosomal proteins.

### Decreased dyskerin expression is associated with poor survival following treatment

**Figure 6:**
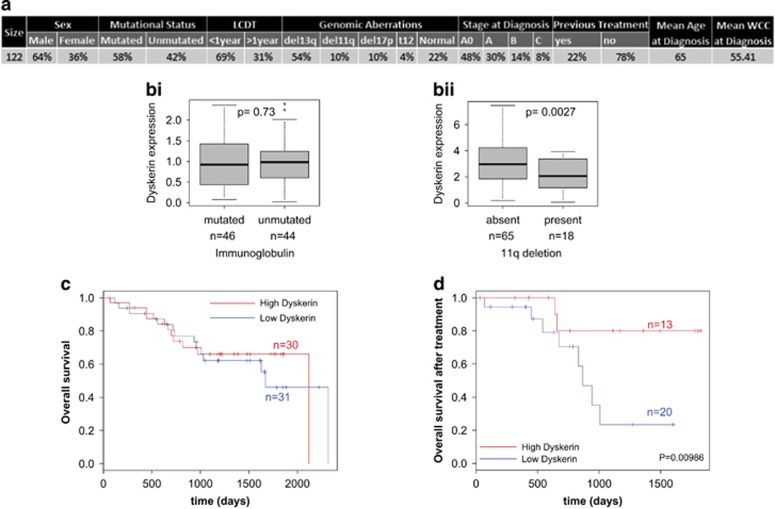
Correlation between dyskerin expression and clinical parameters (**a**) Summary of the clinical data for patients included in the study. (**bi**) Bar plot showing dyskerin protein expression (quantified from western blots) in CLL compared with the IGVH mutational status. The *P*-value was calculated using Student's *t*-test. (**bii**) Bar plot showing dyskerin protein expression (quantified from western blots) in CLL with or without 11q deletion. The *P*-value was calculated using Student's *t*-test. (**c** and **d**) Patients were divided into two groups based on dyskerin expression (low dyskerin *versus* high dyskerin). Survival curves were generated using Kaplan–Meier approximation and significance was estimated by log-rank *P*-value. Impact of dyskerin expression on overall survival was assessed on the whole cohort (**c**) and overall survival following treatment, estimating treatment response (**d**)

It was then important to confirm whether dyskerin expression correlated with previously described markers of CLL and patient survival. Dyskerin levels were examined by western analysis on 98 patient samples and these data were compared with molecular prognostic markers in CLL (Figure 6a,Supplementary Table 2). Although dyskerin expression was independent from IGVH mutational status (Figure 6bi), there was significantly lower expression in patients with 11q deletion (targeting ATM; Figure 6bii), which is associated with poor outcome.

To assess whether dyskerin expression was correlated with survival in CLL, patients were divided into two groups based on their dyskerin expression with a median cutoff. Kaplan–Meier estimation did not show any impact of dyskerin expression on OS (Figure 6c) or progression-free survival (Supplementary Figure 5) in the full cohort. Kaplan–Meier analysis was then performed to assess the patient survival following the first treatment dependent on their dyskerin levels (Figure 6d). The data show that patients with low dyskerin levels had a reduced survival following chemotherapy.

## Discussion

A number of early studies reported reduced ribosomal activity in CLL patients,^48, 49, 50^ which correlated with a reduction in rRNA maturation in this disease.^48, 51, 52, 53^ In this study, the use of translational profiling has allowed the identification of a ‘ribosome-related translational signature' in 34 CLL patients. GO term gene functional analysis of the translational data yielded a set of genes containing ribosomal proteins, translation initiation factors and the pseudouridine synthase *DKC1* (Figure 1). The array data was confirmed by western analysis (Figures 2 and 3), and we provide a link between dyskerin expression and the synthesis of a subset of ribosomal proteins. Thus, cell lines derived from patients with mutations in *DKC1* also have a reduced expression of these proteins (Figure 5). Consistent with these changes, there is a reduction in ribosomal maturation and fewer overall ribosomes (Figure 4). Dyskerin is also required for telomerase activity, as it stabilises the telomerase RNA component. Interestingly, a reduction in DKC1 RNA levels in CLL B cells has been shown previously, and the data suggested that this correlated with telomeric changes in B-CLL.^54^ However, our data show that in PB CLL B cells there is a reduction in DKC1 protein expression in a subset of patient samples (Figure 2bi and ii) and no correlation with telomere length was identified (data not shown).

Importantly, our data suggest that dyskerin expression correlates with poor OS following chemotherapy (Figure 6). We hypothesise that low *DKC1* expression leads to an imbalance in ribosomal proteins and when entering lymph nodes, these changes are likely to influence response of these B cells to the microenvironment. It has been shown previously that mutations in individual ribosomal proteins, for example, RPL38, although not affecting global protein synthesis rates affect transcript-specific translational control.^55^ Therefore, we speculate that in CLL the decrease in *DKC1* expression, leading to reduced synthesis of subsets of ribosomal proteins selectively alters the translatome and that this is in turn is associated with tumorigenesis. In this way, PB CLL B cells fits within a group of disorders terms ribosomopathies that are characterised by defects in ribosomal proteins or biogenesis factors.^17, 55, 56, 57, 58, 59, 60, 61^ In these disorders, patients usually present with hypoproliferative defects such as BM failure; however, with increased age they develop cancers, which are associated with a hyperproliferative state,^16^ in part due to altered translation of subsets of mRNAs. For example, in T-ALL it has been shown that the defective ribosomes cause a reduction in translational fidelity,^55^ whereas in patients with X-linked dyskeratosis congenita initiation of translation of p53 mRNA via its internal ribosome entry segment is impaired.^56^

The identification of somatic mutations in ribosomal proteins that are associated with cancer has refocused the identity of the ribosome in some malignancies from a passive recipient of aberrant signalling cues to a misfunctional cellular machinery.^62^ Despite evidence that these defects are linked to malignant transformation,^17, 60, 63, 64, 65^ the mechanisms by which these ‘cancerous ribosomes' participate in oncogenesis are still not fully understood. However, we propose that in PB CLL the ribosome deficiency is initially linked to a resting phenotype and additional oncogenic events confer a more aggressive phenotype.

## Materials and Methods

### Antibodies

Antibodies were purchased from following companies: Cell Signaling (Danvers, MA, USA) – eIF2 alpha (cs9722), eIF4B (cs3592), eIF4E (cs9742), RPL7a (cs2415), RPS6 (cs2217) and tubulin (cs2146) all used at 1 : 1000; Abcam (Cambridge, UK) – eIF4A1 (ab31217) fibrillarin (ab4566), nucleolin (ab13541), RPL6 (ab50907), RPL19 (52028), RPS16 (ab26159) and RPS23 (ab57644) all used at 1 : 1000; GeneTex (Hsinchu City, Taiwan) – RPL9 (114728) and RPL15 (101830) both used at 1 : 1000; *DKC1* (Santa Cruz, Dallas, TX, USA, sc48794, 1 : 1000); RPS8 (Abgent, San Diego, CA, USA, AP8831a, 1 : 1000); and actin (Sigma-Aldrich, St. Louis, MO, USA, A5441, 1 : 10000).

### Cell culture

Epstein–Barr transformed lymphoblatoid cell lines were derived from dyskeratosis congenita patients using standard methods and were grown in RPMI+20% FBS. These cell lines have been described elsewhere;^24, 25^ however, all three had missense mutations in the *DKC1* gene giving rise to the amino-acid substitutions p.Ala353Val (DC1107), p.Gly402Glu (DC3247) and p.Leu72Tyr (MDH470). Control GM01953 cells were grown in RPMI1640 complemented with 15% FBS.

### Sample collection and purification

CLL samples were collected under Ethical Approval by the REC Committee and under sponsorship from the University Hospitals of Leicester (Study UHL 08654/REC 6978: Target Identification in Haematological Disorders). PB, collected in lithium/heparin collection tubes, was taken from consenting CLL patients attending a weekly haematology clinic at Leicester Royal Infirmary, UK. None of the patients had undergone treatment in the last 3 months. Cycloheximide solution was added directly to the whole blood immediately after it was taken, to a final concentration of 100 *μ*g/ml, and mixed by inversion. The samples were kept on ice during transfer before processing. Normal donor blood products were purchased from the National Blood Service of the National Health Service. The product consisted of leukoreduction cones produced after routine donor platelet pharesis procedures containing white blood cells. Mononuclear cells from CLL patients or normal donor were purified through ficoll gradient. Normal B cells were further purified by positive selective against CD20 using Miltenyi Biotech GmbH (Bergisch Gladbach, Germany) CD20 beads and LS columns following supplier protocol. Purity was checked by flow cytometry using CD45, CD3 and CD19 antibodies.

### Immunofluorescence

The suspension cells were incubated with freshly prepared 4% paraformaldehyde for 10 min at room temperature. A total of 50 × 105 cells were then directly applied to glass slides, as a drop suspension of 3% BSA in PBS. When near to dryness, the cells washed twice in PBS. The cells were permeabilised by incubation with 0.1% Triton X-100 in PBS for 10 min at room temperature and blocked by incubation for 30 min at room temperature with 3% BSA in PBS. The cells were then incubated with primary antibody diluted in 3% BSA/PBS for 1 h at room temperature for fibrillarin and overnight at 4 °C for dyskerin. The cells were then incubated in either Alexa Fluor 568-conjugated goat anti-rabbit IgG or Alexa Fluor 488-conjugated goat anti-mouse IgG (Invitrogen, Thermo Fisher Scientific, Waltham, MA, USA) diluted 1 : 200 in 3% BSA/PBS for 1 h. All images were acquired using a Zeiss LSM510 (Cambridge, UK) meta laser scanning confocal microscope.

### SDS-PAGE and western blotting

Cell lines extracts were prepared as described previously.^26^ Pellets were lysed in lysis buffer at 96 °C before sonication and subjected to protein quantification by BCA assay. Protein extracts were subjected to electrophoresis on SDS-polyacrylamide gels followed by transfer to polyvinylidene difluoride membrane (Millipore, Darmstadt, Germany). Proteins were detected with the relevant antisera using chemiluminescent reagents.^27^

### Sucrose density gradient centrifugation and RNA detection

Sucrose density gradient centrifugation was used to separate ribosomes into polysomal and subpolysomal forms. Gradients were then fractionated with continuous monitoring at 254 nm and RNA was isolated from each fraction as described previously.^28^

### rRNA maturation

For the detection of the different rRNA species, the northern blotting protocol was slightly modified. An amount of 500 ng of total RNA was run in a RNA gel and transferred to a zeta probe membrane overnight. The probe consisted of oligonucleotides specific for the internal transcribed spacer (ITS)-1 (probe ITS1: CCTCGCCCTCCGGGCTCCGTTAATTGATC) and ITS2 (probes ITS2d/e: GCGCGACGGCGGACGACACCGCGGCGTC and ITS2b: CTGCGAGGGAACCCCCAGCCGCGCA) regions. The primers were end labelled with 1 *μ*l of T4 PNK buffer, 50 pmol of each probe, 10 units of T4 PNK and 1.5 *μ*l of 32P *γ*-ATP for 30 min at 37 °C. The probes were cleaned up with G25 sepharose columns and hybridised overnight at 55 °C. Northern blots were exposed to an imaging plate (Fujifilm, Tokyo, Japan) overnight, then visualised on a molecular imager (Bio-Rad, Hercules, CA, USA).

### RNA analysis

Northern analysis of RNA isolated from sucrose density gradients was performed as described previously.^28^ Radiolabelled DNA hybridisation probes were generated using the RadPrime kit according to the manufacturer's instructions (Invitrogen). Northern analysis on total RNA samples obtained following were performed as described on at least three independent occasions. For RT-PCR analysis, 1 *μ*g of total RNA was reverse transcripted using Superscript III RNase H-Reverse transcriptase (Invitrogen) and random primers in conditions specified by the manufacturer, then PCR was performed on 1 *μ*g of cDNA using 1 pmol of each primer, 2.5 *μ*mol of dNTP mix and 0.5 *μ*l of Taq polymerase (Roche, Basel, Switzerland).

### Preparation of fluorescently labelled cDNA for microarray hybridisation and data analysis

The human cDNA microarrays contained a set of ~10 000 human cDNA clones, manufactured in MRC Toxicology Unit Leicester. Fluorescently labelled DNA probes were generated from equal proportions of RNAs (~7 *μ*g) of pooled polysomal fractions (fractions 1–5) and pooled non-polysomal fraction (fractions 6–11) or with fixed amount of universal commercial RNA control (UniRNA Supplier, Stratagene, San Diego, CA, USA). Microarray slides were scanned using a GenePix 4200A microarray scanner and GenePix Pro 5.1 software (Axon Instruments, Union City, CA, USA).

### Patients and cells for studies *in vitro*

Patients provided written informed consent in accordance with Ethics Committee approvals and the Declaration of Helsinki. Heparinized PB mononuclear cells were obtained from patients attending clinics at the Southampton General Hospital or the Royal Berkshire Hospital (both UK; Supplementary Table S1). Diagnosis of CLL was according to the IWCLL-NCI 2008 criteria^29^ and the monoclonal B-lymphocyte population in the PB had a typical IgM+IgD+ CLL phenotype in all circumstances.^30^ The vast majority of samples were obtained before treatment. Where treatment for CLL had taken place, this was at least 6 months before sample collection. IGHV usage and homology to germline, expression of cell surface CD5, CD19, CD23 and CD38, and ZAP70 were determined as previously described.^31^ sIgM signalling capacity was determined by measuring the percentage of cells with increased intracellular Ca2+ following stimulation with soluble goat F(ab′)2 anti-IgM and using a cutoff value of ⩾5% responding cells to define samples as sIgM responsive as previously described.^31^

Cryopreserved CLL cells were recovered and rested for 1 h at 37 °C before use. CLL cell viability determined by trypan blue exclusion was ⩾90% and the median proportion of CD5+CD19+ CLL cells was 95% (range 62–99%). For sIg stimulation, samples were incubated with bead-bound goat F(ab′)2 anti-human IgM or control antibodies.^32^

### Analysis of microarray data

GenePix Pro 5.1 was used to quantify fluorescence intensities for individual spots on the microarray. Microarray data were processed in the R-CRAN platform using the Limma package.^33^ The data were background corrected and normalised between arrays against the universal RNA control using a quantile approach. Technical replicates were averaged for each patient. The log2 transformed ratios of polysomal over subpolysomal signal were then subjected to a panel of four different statistical approaches: (1) « Limma » based on model fitting in the Limma package;^33^ (2) « Rankprod » based on ranking analysis in the RankProd package;^34, 35^ (3) « SAM » based on non-parametric approach;^36^ (4) and « *t*-test » based on a basic Student's *t*-test. Genes identified as significantly deregulated in each list were filtered to select only genes identified in at least three of the four tests as significantly deregulated. This final list was analysed using gene ontology (GO) annotations with DAVID^37, 38^ for gene functional classification using high stringency parameters and enrichment score >1.3 corresponding to a *P*-value <0.05. Heatmap was produced using MeV 4.0 and network visualisation was implemented with CytoScape v3.2.1.^66^

### Image analysis

Image quantification was performed using ImageJ software. (National Institutes of Health, Bethesda, MD, USA).

## Abbreviations

CLL: chronic lymphocytic leukaemia
PB: peripheral blood
LN: lymph nodes
BM: bone marrow
IGVH: immunoglobulin heavy-chain variable region
eIF: eukaryotic initiation factor
BCR: B-cell receptor
T-ALL: T-cell acute lymphoblastic leukaemia
RPS: ribosomal protein small
RPL: ribosomal protein large
ITS: internal transcribed spacer
