# Supplementary material for: A ribosome-related signature in peripheral blood CLL B cells is linked to reduced survival following treatment
Source: Cell Death Dis. 2016 Jun 2;7(6):e2249–. doi: 10.1038/cddis.2016.148 (PMC5143378; doi:10.1038/cddis.2016.148)
Supplement: Supplementary Table 2 [file cddis2016148x2.ppt]

## Slide 1
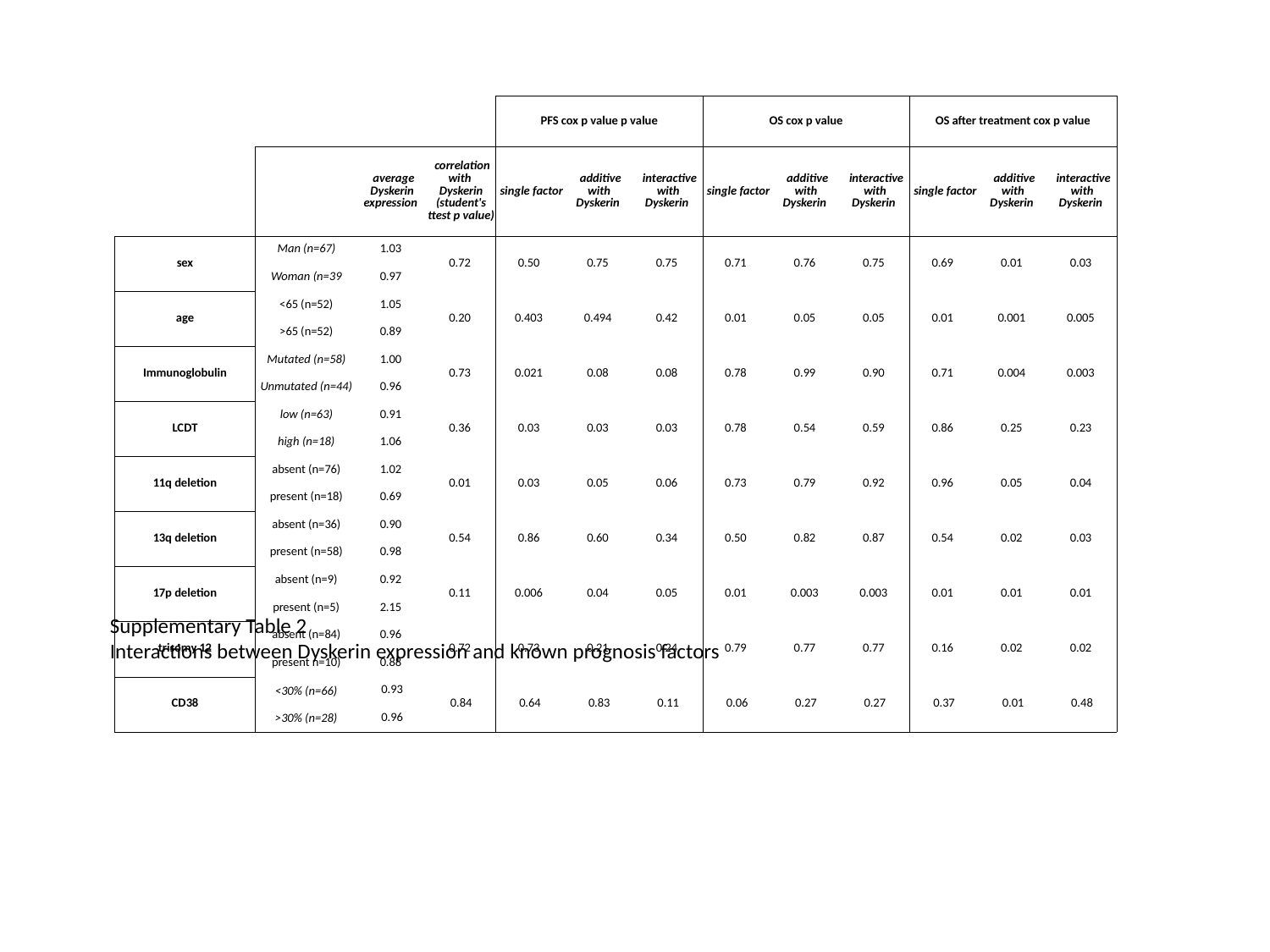

| | | | | PFS cox p value p value | | | OS cox p value | | | OS after treatment cox p value | | |
| --- | --- | --- | --- | --- | --- | --- | --- | --- | --- | --- | --- | --- |
| | | average Dyskerin expression | correlation with Dyskerin (student's ttest p value) | single factor | additive with Dyskerin | interactive with Dyskerin | single factor | additive with Dyskerin | interactive with Dyskerin | single factor | additive with Dyskerin | interactive with Dyskerin |
| sex | Man (n=67) | 1.03 | 0.72 | 0.50 | 0.75 | 0.75 | 0.71 | 0.76 | 0.75 | 0.69 | 0.01 | 0.03 |
| | Woman (n=39 | 0.97 | | | | | | | | | | |
| age | <65 (n=52) | 1.05 | 0.20 | 0.403 | 0.494 | 0.42 | 0.01 | 0.05 | 0.05 | 0.01 | 0.001 | 0.005 |
| | >65 (n=52) | 0.89 | | | | | | | | | | |
| Immunoglobulin | Mutated (n=58) | 1.00 | 0.73 | 0.021 | 0.08 | 0.08 | 0.78 | 0.99 | 0.90 | 0.71 | 0.004 | 0.003 |
| | Unmutated (n=44) | 0.96 | | | | | | | | | | |
| LCDT | low (n=63) | 0.91 | 0.36 | 0.03 | 0.03 | 0.03 | 0.78 | 0.54 | 0.59 | 0.86 | 0.25 | 0.23 |
| | high (n=18) | 1.06 | | | | | | | | | | |
| 11q deletion | absent (n=76) | 1.02 | 0.01 | 0.03 | 0.05 | 0.06 | 0.73 | 0.79 | 0.92 | 0.96 | 0.05 | 0.04 |
| | present (n=18) | 0.69 | | | | | | | | | | |
| 13q deletion | absent (n=36) | 0.90 | 0.54 | 0.86 | 0.60 | 0.34 | 0.50 | 0.82 | 0.87 | 0.54 | 0.02 | 0.03 |
| | present (n=58) | 0.98 | | | | | | | | | | |
| 17p deletion | absent (n=9) | 0.92 | 0.11 | 0.006 | 0.04 | 0.05 | 0.01 | 0.003 | 0.003 | 0.01 | 0.01 | 0.01 |
| | present (n=5) | 2.15 | | | | | | | | | | |
| trisomy 12 | absent (n=84) | 0.96 | 0.72 | 0.73 | 0.21 | 0.24 | 0.79 | 0.77 | 0.77 | 0.16 | 0.02 | 0.02 |
| | present n=10) | 0.88 | | | | | | | | | | |
| CD38 | <30% (n=66) | 0.93 | 0.84 | 0.64 | 0.83 | 0.11 | 0.06 | 0.27 | 0.27 | 0.37 | 0.01 | 0.48 |
| | >30% (n=28) | 0.96 | | | | | | | | | | |
Supplementary Table 2
Interactions between Dyskerin expression and known prognosis factors
