# Supplementary material for: A ribosome-related signature in peripheral blood CLL B cells is linked to reduced survival following treatment
Source: Cell Death Dis. 2016 Jun 2;7(6):e2249–. doi: 10.1038/cddis.2016.148 (PMC5143378; doi:10.1038/cddis.2016.148)
Supplement: Supplementary Figures [file cddis2016148x3.pdf]

Sbarrato_Supplementary_Fig1

A

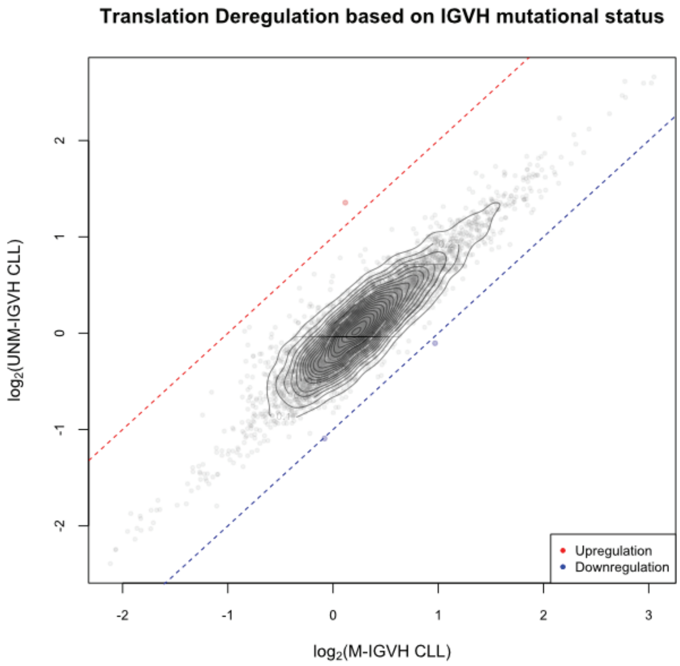

B

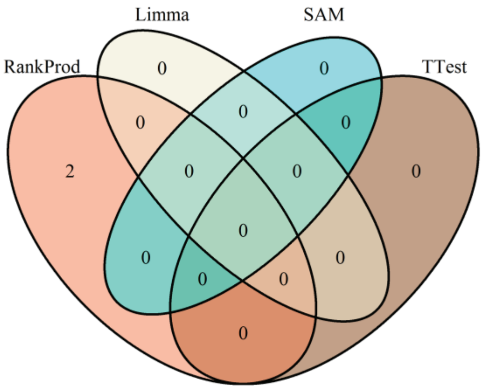

Supplementary Figure 1. Translatome analysis of CLL patients based on IGVH mutational status
A) Profile for the translatome of unmutated IGVH CLL versus mutated IGVH CLL patients. Processed translatome data (log2 ratios for each gene averaged across patients) were plotted for mutated IGVH CLL patients (x axis) versus unmutated IGVH CLL patients. Grey, red and blue dots represent gene values for unchanging, upregulation and dowregulation respectively. Red and blue dotted line represent the limit for fold change equal to 2 and 0.5 respectively. Contour plot was superimposed to highlight general shape of translatome (dark lines). **B)** 4-way Venn Diagram representing the overlap of significantly identified genes for deregulation between unmutated and mutated IGVH CLL patients in the four different statistical tests used: RankProd, Limma-eBayes, SAM and ttest.

Sbarrato_Supplementary_Fig2

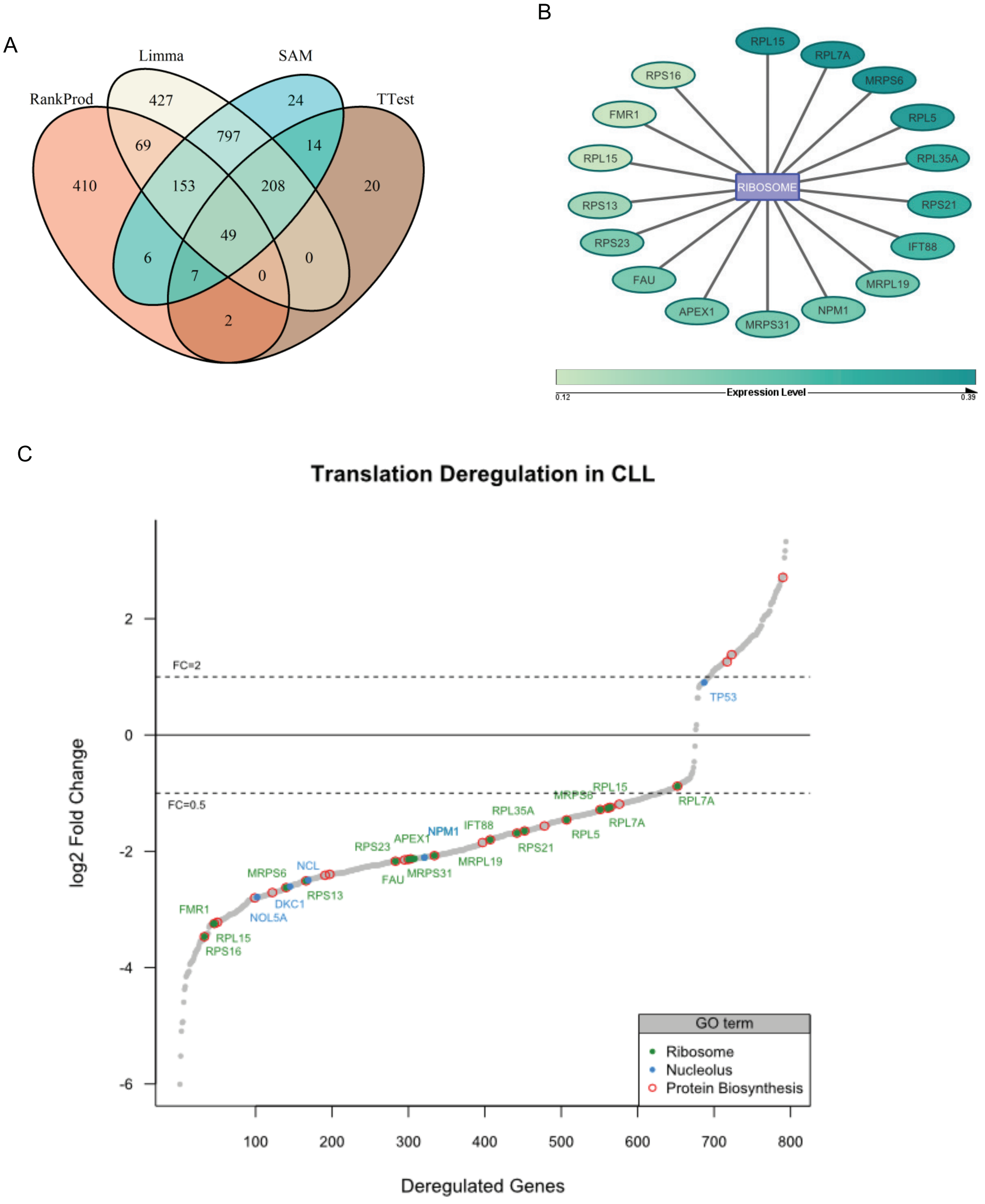

Supplementary Figure 2. Translatome analysis of CLL patients versus CTRL B cells.

A4-way Venn Diagram representing the overlap of significantly identified genes for deregulation between CLL patients and CTRL B cells in the four different statistical tests used: RankProd, Limma-eBayes, SAM and ttest. **B)** Network analysis for GO term “Ribosome” among the significant genes identified in at least 3 out of four statistical tests. Colour gradient represents the expression level of the translatome for each gene. **C)** Enrichment of GO Terms “Ribosome”, “Protein Biosynthesis” and “Nucleolus” among the significant genes identified in at least 3 out of four statistical tests. Genes were ranked and plotted by fold change between CLL patients and CTRL B cells.

Sbarrato_Supplementary_Fig3

A

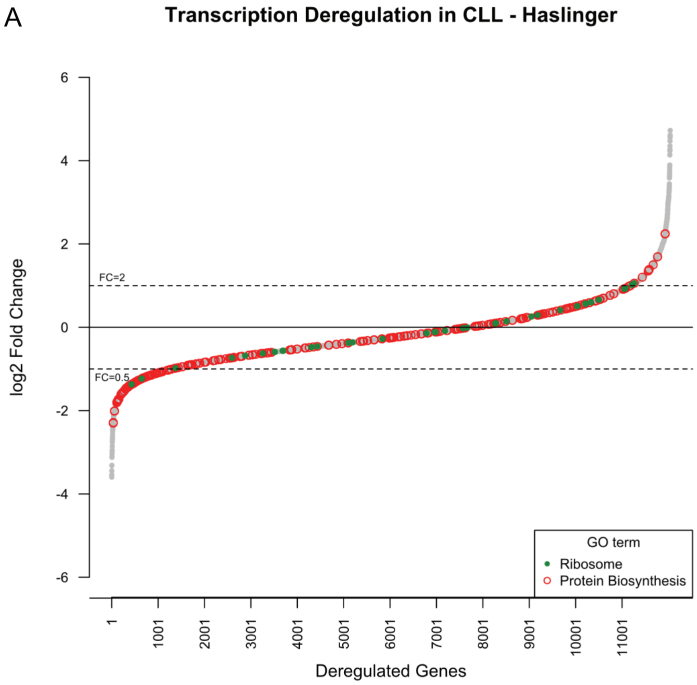

B

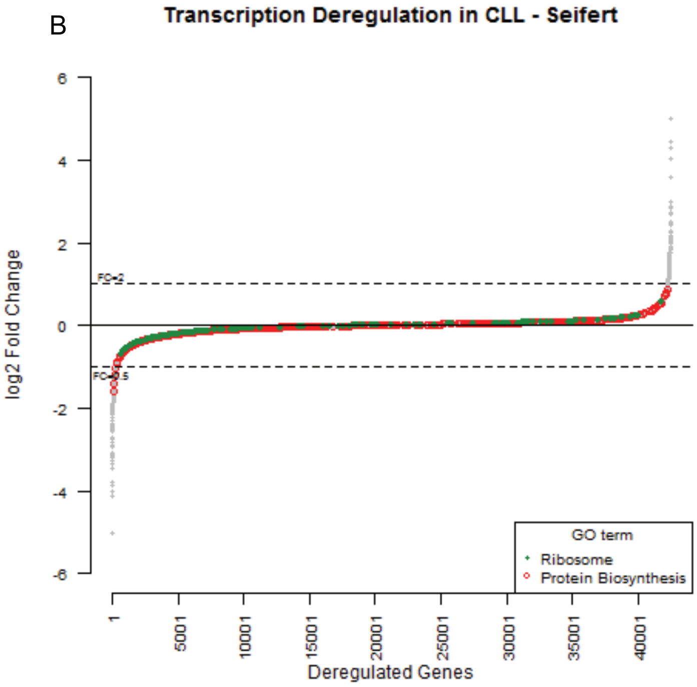

Supplementary Figure 3. Transcriptome analysis of two published datasets for CLL patients versus CD19+ or CD5+ B cells Controls. A) GO term analysis of CLL Transcriptome for GO Terms “Ribosome” and “Protein Biosynthesis” among all genes from published dataset Haslinger between CLL patients and CD19+ CTRL B cells [32]. Genes were ranked and plotted by fold change between CLL patients and CD19+ CTRL B cells.
B) GO term analysis of CLL Transcriptome for GO Terms “Ribosome” and “Protein Biosynthesis” among all genes from published dataset Seifert between CLL patients and CD5+ CTRL B cells [57]. Genes were ranked and plotted by fold change between CLL patients and CD5+ CTRL B cells.

Sbarrato_Supplementary_Fig4

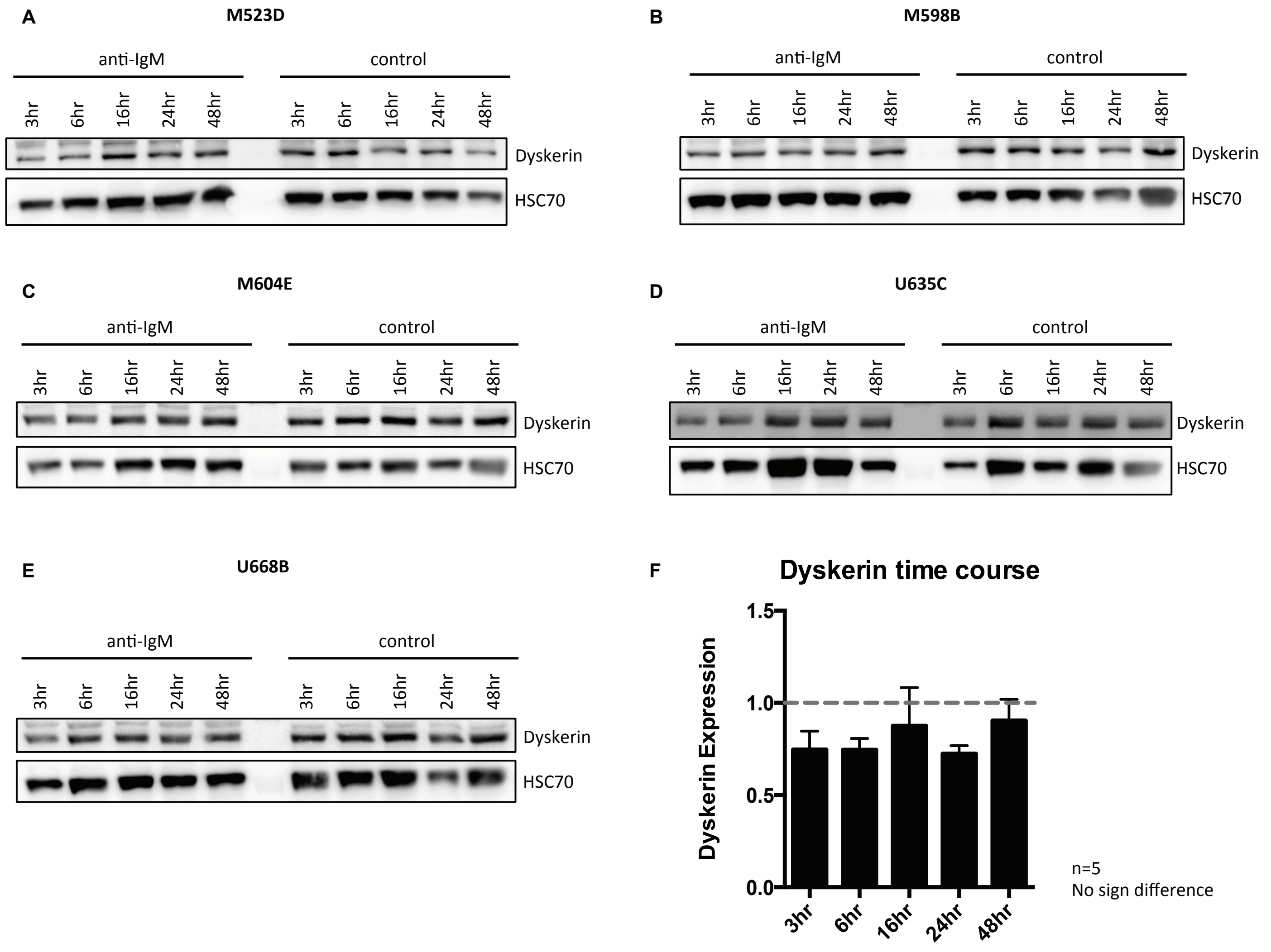

Supplementary figure 4: **Dyskerin expression in IgM stimulated CLL cells.** CLL cells from five different patients were incubated in the presence of anti-IgM coupled Dynabeads or control antibodies for the indicated time. Western blots were performed to show expression of Dyskerin after 3, 6, 16, 24 and 48hrs (A-E). Dyskerin expression was quantified and normalized to HSC70 before anti-IgM Dyskerin expression was made relative to control bead incubation for each time point. (F) Graph represents Dyskerin expression following BCR stimulation with control bead Dyskerin expression for each time point set to 1, bars (±SEM) represent the mean expression, n=5 no significant difference.

Sbarrato_Supplementary_Fig5

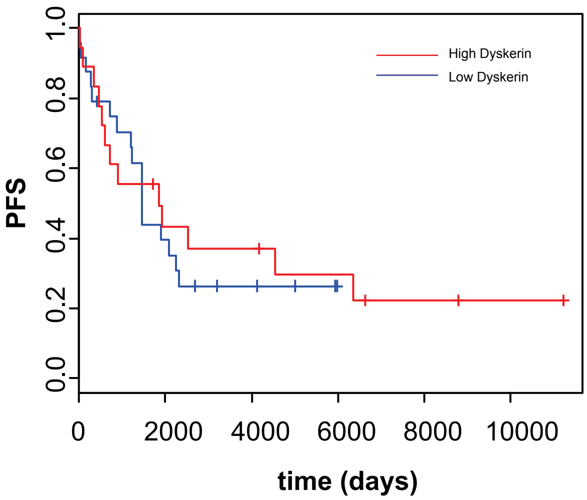

Supplementary Figure 5. Correlation between dyskerin expression and progression free survival. Patients were divided into two groups based on dyskerin expression (low dyskerin vs high dyskerin) as in Figure 6B. Impact of dyskerin expression on progression free survival was generated using Kaplan Meyer approximation and significance was estimated by Log-Rank p value.
